# Regulation and Mechanism of miR-518d through the PPAR*α*-Mediated NF-*κ*B Pathway in the Development of Gestational Diabetes Mellitus

**DOI:** 10.1155/2020/7019597

**Published:** 2020-10-17

**Authors:** Hui Qiu, Xuemin Liu, Shenshen Yao, Jiaren Zhou, Xue Zhang, Juan Du

**Affiliations:** Department of Gynaecology and Obstetrics, Shengjing Hospital of China Medical University, Shenyang, Liaoning 110004, China

## Abstract

**Objectives:**

To observe the role of miR-518d in pregnant women with gestational diabetes mellitus (GDM) and its adjusting effects on PPAR*α* and to explore the regulatory mechanisms of the NF-*κ*B pathway in the development and progression of GDM.

**Methods:**

Placenta tissues and peripheral plasma were obtained from pregnant women with normal pregnancy and GDM, respectively, followed by the detections of miR-518d contents by RT-PCR and the expression levels of inflammatory factors using ELISA. Human placenta trophoblast cells (HTR8/SVneo) were cultured under the conditions of physiological glucose (PG group) and high glucose level (HG group). Cells in the HG group were transfected with miR-518d control, mimics, and inhibitors and were separately administered with a PPAR*α*-specific antagonist (GW6471) and PPAR*α* siRNA, and cells were divided into the following groups: HG+miR-518d control group (HGNC group), HG+miR-518d mimic group (HGM group), HG+miR-518d inhibitor group (HGI group), HGI+PPAR*α* antagonist group, and HGI+PPAR*α* siRNA group. The relative expression levels of miR-518d, PPAR*α*, and its downstream genes and NF-*κ*B signalling pathway-related genes were detected by RT-PCR and Western blotting. The contents of inflammatory factors were examined by Western blotting. A dual-luciferase report assay was performed to validate the correlations between miR-518d and PPAR*α*. In this study, mouse GDM models were established to further prove the previous hypothesis with an *in vivo* experiment. A total of 40 C57BL/6J mice were randomly divided into the following groups: normal diet group (Control_Ms_), GDM group (GDM_Ms_ group), GDM+miR-518d antagomir group, and GDM+miR-518d antagomir+PPAR*α* antagonist group. The mouse model of GDM was established by feeding with combined high-sugar and high-saturated fat diet and injecting streptozotocin (STZ) after 15-day feeding. Female and male mice were cocaged in the number ratio of 2 : 1, and the evidence of vaginal suppository detected in female mice was marked as D0 of pregnancy. The contents of total cholesterol (CH), triglyceride (TG), fast glucose, and insulin (INS) were examined using ELISA, followed by the evaluation of insulin resistance (IR). The related expression levels were also detected with the above methods shown in the previous cell culture.

**Results:**

miR-518d has a high expression level in placentas with GDM. As the target gene of miR-518d, PPAR*α* was downregulated with the increased levels of miR-518d. When GDM occurs, inflammatory responses were elevated, stimulating the nuclear transport process of NF-*κ*B. Activated NF-*κ*B triggered the phosphorylation of IKK*β* and I*κ*B*α*.

**Conclusions:**

High expression of miR-518d was observed in the development of GDM. In this study, we validated that miR-518d negatively regulates the expression of PPAR*α* and triggers the nuclear transport process of NF-*κ*B and phosphorylation of pathway-associated proteins leading to an inflammatory response and the development of GDM.

## 1. Introduction

Gestational diabetes mellitus (GDM) refers to a disorder of abnormal glucose metabolism that occurs or is first discovered during pregnancy and is one of the most common complications during pregnancy [1]. With the changes in the lifestyle and dietary pattern, the incidence of obesity during pregnancy and GDM has increased year by year. Several social surveys have demonstrated that about 8% of pregnant women were found to have GDM globally; however, the prevalence might be varied due to the different social and economic development and the diagnostic capacity in various countries [2]–[3]. GDM can also lead to serious, even fatal complications for both pregnant women and offspring; it has been found that GDM not only significantly increases the incidence of preeclampsia, macrosomia, dystocia, and fatal distress but also is closely related to many adverse pregnancy outcomes such as miscarriage, intrauterine fatal death, and fatal malformations [3]–[4]. The mechanism of GDM occurrence and development has not been fully clarified, and research on its pathogenesis has important theoretical significance for promoting the clinical treatment of GDM.

MicroRNAs (miRs) are endogenous, single-stranded, non-protein-coding RNAs in a short length of approximately 22 nt and regulate the relative gene expression through binding to the 3′ untranslated region (3′-UTR) of target messenger RNAs (mRNAs) [5]–[6]. Studies have suggested that miRs are involved in GDM, implying that the relevant miRs play essential roles in disease pathogenesis and may act as diagnostic biomarkers in the early development of GDM [6, 7]. MicroRNA-518d (miR-518d) is one of the most important miRNAs which exert high expression in the placenta tissues with GDM. Accumulated evidence has shown that the mRNA and protein expression levels of glucose transporter 1 (GLUT1) and glucose transporter 4 (GLUT4) are negatively correlated with the expression level of miR-518d in placenta tissues with GDM, suggesting that miR-518d affects the glucose transport and insulin sensitivity through inhibiting the expression of GLUT1 and GLUT4. It has also been proven that the peroxisome proliferator-activated receptor-*α* (PPAR*α*) is the direct target of miR-518d and there is a specific binding site on the seed region [8], suggesting that miR-518d may get involved in the progression and development of GDM through the PPAR*α*-mediated pathway.

GDM is a disease mainly characterised by high blood glucose level complicated by chronic inflammatory response. Relatively high inflammatory response in placenta tissues was detected in pregnancies with GDM; hence, determining the expression levels of related inflammatory factors may be crucial for the clinical diagnosis and research on the occurrence and development of GDM [9]. Peroxisome proliferator-activated receptors (PPARs) are a group of nuclear transcriptional factors which are widely expressed in multiple tissues of the body; among three isotypes of PPAR (*α*, *β*, and *γ*), the expression of PPAR*α* is mainly observed in the placental tissues in which it regulates the lipid metabolism and inflammatory response [10, 11]. Nuclear factor-kappa B (NF-*κ*B) is a family of transcription factor proteins, which are involved in cell growth, adhesion, and inflammation. NF-*κ*B can interact with tumour necrosis factor alpha (TNF-*α*) to form a positive feedback loop of low-grade inflammation and increase insulin resistance in GDM and thus promote the development of GDM. Studies have proven that the ligand of PPAR*α* could negatively regulate the NF-*κ*B pathway and activator protein 1 (AP-1) activation triggering anti-inflammatory response [12]; meanwhile, activating and upregulating PPAR*α* can also suppress the LPS-induced NF-*κ*B signalling pathway in macrophages [13]. Taken together, the ligand of PPAR*α* can repress NF-*κ*B activity leading to the inhibition of inflammatory responses, suggesting that PPAR*α* is correlated with the NF-*κ*B pathway. In this study, we detected the expression levels of miR-518d, PPAR*α*, and relative inflammatory factors in plasma and placental tissues of pregnant women followed by cell culture and animal experiments to explore the regulatory mechanism of miR-518d through the PPAR*α*-mediated NF-*κ*B pathway in the development and progression of GDM.

## 2. Materials and Methods

### 2.1. Patients and Placenta Tissue Preparation

Human placenta tissues were obtained from 36-42-week pregnant women from Shengjing Hospital of China Medical University (Shenyang, China), from Jan 2016 to Dec 2018. Placenta tissues were obtained from 60 pregnant women with normal pregnancy (control group) and another 60 patients with GDM (GDM group). All subjects with a history of other complications were excluded from this study. The principle of the Declaration of Helsinki was conducted strictly. The study was approved by the Local Ethics Review Board of Shengjing Hospital of China Medical University, and informed consent was obtained from all participants.

1 cm^3^ of placenta tissues was obtained from the above groups of pregnant women and was frozen in the liquid nitrogen directly for the following tests in this study. 2 mL of peripheral plasma was harvested from all participants followed by centrifugation at 4000g, at 4°C for 10 minutes, and the serum was stored at -80°C.

### 2.2. Human Placenta Trophoblast Cell Culture

The HTR8/SVneo cell line was purchased from PriCells (Wuhan, China) and was cultured in RPMI 1640 medium (12633012, Gibco, USA) with 10% fetal bovine serum (04-001-1A, BI, USA). Human placenta trophoblast cells were cultured at physiological glucose level (5 mmol/L) (PG group) and high glucose level (25 mmol/L) (HG group). HG cells were transfected with miR-518d control (HGNC group), mimics (HGM group), and inhibitors (HGI group) which were purchased from Jijia Biotech (Shenyang, China). The transfection processes were conducted with Lipofectamine 3000 (L3000015, Invitrogen, USA). To explore the regulatory mechanism of the PPAR*α* signalling pathway in correlation with miR-518d, a PPAR*α*-specific antagonist (GW6471) (G5045, Sigma-Aldrich, USA) was added into HGI cells. Cultured cells were collected freshly for the following detections of mRNA and protein expression levels and were frozen in the liquid nitrogen for later use.

### 2.3. GDM Mouse Model Establishment

The 4-week-old female and male C57BL/6J mice were purchased from Vitalriver (Beijing, China), and the study has been authorised by the Experimental Animal Welfare and Ethics Committee of China Medical University (IACUC no. 2019097). Mice were randomly divided into two groups: mice in the Control_Ms_ group were given the normal diet and the high-sugar and high-saturated fat combined diet was applied to the mice in the GDM_Ms_ group. After 12-week feeding, a certain concentration (35 mg/kg) of 0.25% streptozotocin (STZ) was administered to the GDM_Ms_ group. The value of random blood glucose level higher than 16.7 mmol/L was detected at 3 days postinjection, indicating that the GDM mouse models were established successfully. Female and male mice with GDM were caged in a 2 : 1 number ratio, and the vaginal suppository or vaginal secretion smear microscopy was checked on the 2^nd^ day after cocaging. The evidence of vaginal suppository detected in female mice was counted as the 0 day of pregnancy. 100 nM of the miR-518d antagomir and the combined administration of the miR-518d antagomir and GW6471 were injected to the mice of the GDM_Ms_ group via tail veins, respectively, to explore the regulatory mechanism of miR-518d on the development and progression of GDM.

### 2.4. Pathological Tests on Mouse Models

Body weights of mice were examined and recorded in the morning after 12-hour fasting at 0, 3, 10, and 18 days of pregnancy. Mice were fasted for 16 hours prior to the intraperitoneal glucose tolerance test (IPGTT) at the 18^th^ day of pregnancy, and a certain amount of 20% glucose solution (2 g glucose/kg) was injected intraperitoneally. The blood glucose levels were then measured at 0, 15, 30, 60, 90, and 120 minutes after glucose injection, and the obtained data were analyzed later. Plasma samples were collected from the tail veins after 12-hour fasting in the morning of the 18^th^ day of pregnancy. The contents of total cholesterol (CH), triglyceride (TG), fast blood glucose (FBG), and insulin (INS) of each group were measured, and then, the homeostatic model assessment of insulin resistance (HOMA-IR) was calculated based on the obtained data.

### 2.5. Fluorescence *In Situ* Hybridization (FISH)

4 *μ*m of paraffin-embedded tissue slices was mounted onto an adhesive slide and heated for 30 min at 80°C, followed by the deparaffinisation process at 68°C for 15 min. Slices were then rinsed with absolute ethanol twice at room temperature (5 min/time). Straight afterwards, the permeation process was carried out in deionised water at 90°C for 40 min, followed by washing with distilled water for 3 minutes. Enzymatic digestion was performed at 37°C for 10-40 min, and slices were dehydrated with gradient ethanol for 2 min each and then dried at room temperature. Slides were placed into pepsin solution at 85°C for 5 minutes for denaturation, and the probe was added on the slides and a coverslip was placed to avoid the formation of air bubbles at 42°C for 2 hours for hybridisation. DAPI counterstaining was applied onto the slides for 10 minutes protected from light. Analysis at the target area was observed under a fluorescence microscope.

### 2.6. Enzyme-Linked Immunosorbent Assay (ELISA)

The expression levels of inflammatory factors of NF-*κ*B (SEB824Hu, USCN, USA), TNF-*α* (SEA133Hu, USCN, USA), interleukin-1 beta (IL-1*β*) (SEA563Hu, USCN, USA), interleukin-6 (IL-6) (SEA079Hu, USCN, USA), and cytochrome C oxidase subunit II (COX-2) (SED284Hu, USCN, USA) in women placenta tissues, peripheral plasma of pregnant women, and the HTR8/SVneo cell line were detected. In the mouse models, the ELISA assay was performed to examine the contents of CH (CEB701Ge, USCN, USA), TG (CEB687Ge, USCN, USA), INS (CEA448Mu, USCN, USA), NF-*κ*B (SEB824Mu, USCN, USA), TNF-*α* (SCA133Mu, USCN, USA), IL-1*β* (SEA563Mu, USCN, USA), IL-6 (SEA079Mu, USCN, USA), and COX-2 (SED284Mu, USCN, USA) in the placental peripheral plasma in mice. The manufacturer's instructions were strictly followed in this study. Briefly, standards and samples were added into the plates, and then, the plates were incubated at 37°C for one hour. 100 *μ*L of detection reagents A and B was then applied to each well, respectively, after rinsing three times with washing buffer. Then, a 30-minute incubation was carried out. Later, plates were incubated for another 15 minutes with the addition of TMB substance solution and were kept away from light. Without rinse, stop solution was applied to all the wells, and it was notable that the solution turned into yellow immediately. Within 10 minutes after the colour changed, the OD values were determined with a microplate reader at a wavelength of 450 nm.

### 2.7. Real-Time Polymerase Chain Reaction (RT-PCR)

Total RNA was extracted from placentas using the CellAmp™ Direct RNA Prep Kit for RT-PCR (3732, Takara, Japan) following the manufacturer's instructions; then, it was reversely transcribed into cDNA. The expression of COX-2, IL-1*β*, TNF-*α*, NF-*κ*B, and PPAR*α* was measured in Exicycler 96 Real-Time Quantitative Thermal Block (Bioneer, Daejeon, Korea) using TB Green® Premix Ex Taq™ II (RR820A, Takara, Japan). RNA concentration was measured using Nanodrop 2000. cDNA was reverse transcribed using a PrimeScript™ RT Reagent Kit (RR037A, Takara, Japan), and real-time PCR was performed using Probe qPCR Mix (RR392A, Takara, Japan) in the 7500 Fast Real-time PCR system (Applied Biosystems). Fold change of mRNA expression was calculated using the 2^-*ΔΔ*Ct^ method. For the miR-518d, total RNA was reversely transcribed using the TagMan MicroRNA Reverse Transcription Kit (Applied Biosystems, UK) following the manufacturer's instructions. miR-518d expression was determined by using TagMan assays (Applied Biosystems, UK).

### 2.8. Dual-Luciferase Reporter (DLR) Assay

To validate whether PPAR*α* is the target gene of miR-518d, HTR8/SVneo cells were cotransfected with plasmid containing miR-518d control or mimics and firefly luciferase reporter constructs containing wild-type (WT) and mutated (MUT) 3′-UTR of PPAR*α* using Lipofectamine 3000 (L3000015, Invitrogen, USA), respectively. The DLR assay was performed using the Double-Luciferase Reporter Assay Kit (abx098134, Abbexa, UK). Instructions were strictly followed. Briefly, the growth medium from cultured cells was eliminated, and cells were rinsed with PBS buffer. After the removal of all rinse solutions, PLB lysis buffer was added into each culture vessel. The lysate product was transferred to a tube after shaking the vessel for 15 minutes at room temperature. 100 *μ*L of LAR II was added into a luminometer tube, and another 20 *μ*L PLB lysate was transferred into the tube followed by thorough mixing. 100 *μ*L of Stop & Glo® Reagent was dispensed into the tube after measuring the activity of firefly luciferase, and the Renilla luciferase activity was then measured.

### 2.9. Western Blotting

Frozen placenta tissues and collected cells were taken out from liquid nitrogen and lysed with RIPA lysis and extraction buffer (89901, Thermo Fisher Scientific, USA). The manufacturer's instruction was strictly followed. The protein samples were collected from the supernatants after centrifugation. Protein samples were then loaded into SDS-PAGE gels to run for electrophoresis, followed by PVDF membrane transfer and skim milk blocking processes. Postblocking membranes were incubated at room temperature with primary antibodies against PPAR*α* (ab24509, Abcam, USA), CD36 (ab133625, Abcam, USA), acyl-CoA oxidase (ACO) (ab248375, Abcam, USA), uncoupling protein 2 (UCP2) (ab97931, Abcam, USA), NF-*κ*B (ab131546, Abcam, USA), IKK*β* (ab124957, Abcam, USA), p-IKK*β* (ab59195, Abcam, USA), I*κ*B*α* (ab7217, Abcam, USA), and p-I*κ*B*α* (ab133462, Abcam, USA). The primary antibodies against GAPDH (ab181602, Abcam, USA) and histone H3 (ab1791, Abcam, USA) were separately used as loading controls in total cell proteins and nucleus proteins. An HRP-conjugated goat anti-rabbit secondary antibody (ab6721, Abcam, USA) was applied to the membranes after the removal of primary antibodies, followed by an incubation of 1 hour at room temperature. SuperSignal West Pico PLUS Chemiluminescent Substrate (34580, Thermo Fisher Scientific, USA) was used during the grey analysis by using an imaging system.

### 2.10. Statistical Analysis

Statistical analysis was performed by using SPSS 19.0 Statistics (IBM® SPSS, USA). All data were represented as mean ± SD. Student's *t*-test was used for the statistical analysis between groups, and the Wilcoxon test was used in the intragroup comparison.

## 3. Results

### 3.1. Regulatory Effects of miR-518d on Pathological Characteristics in GDM Patients

**Figure 1:**
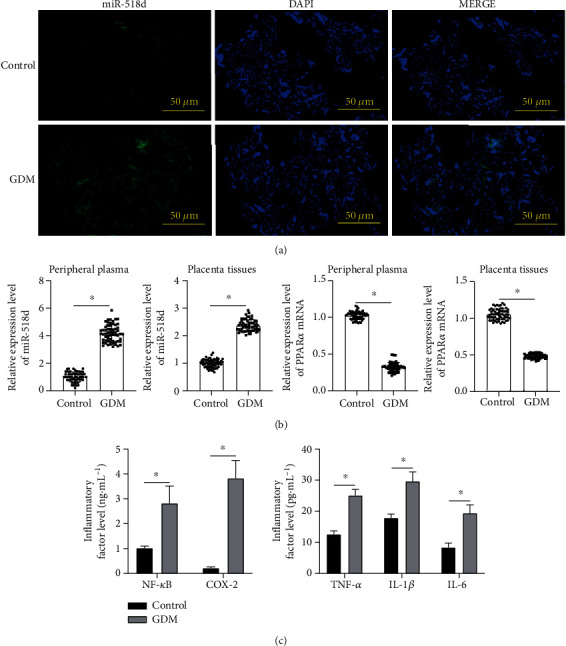
Regulatory effects of miR-518d on pathological characteristics in GDM patients. (a) The expression of miR-518d in placenta tissues of pregnant women observed by the FISH assay (scale bar = 50 *μ*m). (b) Expression levels of miR-518d and PPAR*α* in peripheral plasma and placenta tissues of pregnant women examined by RT-PCR. (c) Contents of inflammatory factors (NF-*κ*B, TNF-*α*, IL-1*β*, IL-6, and COX-2) in peripheral plasma detected by ELISA. All data were represented as mean ± SD. ^∗^*p* < 0.05.

To prove that miR-518d is one of placenta-specific miRNAs, FISH analysis was carried out on paraffin-embedded placental tissue sections; results illustrated the high expression of the miR-518d gene in the placental tissues of GDM patients (Figure 1(a)). Expression levels of miR-518d and PPAR*α* in peripheral plasma and placental tissues were detected using RT-PCR; the upregulation of miR-518d was found in pregnant women with GDM, while PPAR*α* was opposite to the expression of miR-518d (Figure 1(b)). GDM is a disease complicated by chronic inflammatory response, and increased levels of relevant inflammatory factors (NF-*κ*B, TNF-*α*, IL-1*β*, IL-6, and COX-2) in the peripheral plasma of pregnant women's placenta with GDM were detected (Figure 1(c)), suggesting that the inflammatory response is highly correlated with the development and progression of GDM leading to the GDM-associated clinicopathological characteristics and the possible consequences related to pregnancy outcomes.

### 3.2. miR-518d Affects the Inflammatory Responses in the Human Placental Trophoblast Cell Line

**Figure 2:**
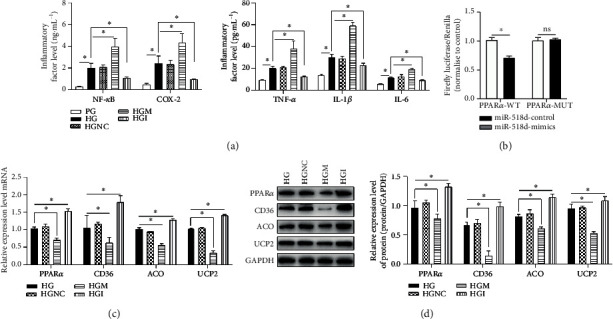
miR-518d affects the inflammatory responses in HTR8/SVneo cells. (a) Levels of relative inflammatory factors (NF-*κ*B, TNF-*α*, IL-1*β*, IL-6, and COX-2) in HTR8/SVneo cells cultured at various conditions detected by ELISA. (b) Validation of the correlation between miR-518d and PPAR*α* detected by the DLR assay. Expression levels of PPAR*α* and its downstream genes (CD36, ACO, and UCP2) examined by (c) RT-PCR and (d) Western blotting. All data were represented as mean ± SD. ^∗^*p* < 0.05.

A human placental trophoblast cell line, HTR8/SVneo, was cultured under the conditions of PG and HG, respectively. Expression levels of inflammatory factors (NF-*κ*B, TNF-*α*, IL-1*β*, IL-6, and COX-2) were increased in the HGM group; with the transfection of miR-518d inhibitors, the relevant inflammatory responses were relieved (Figure 2(a)). To validate whether PPAR*α* is the target gene of miR-518d, the dual-luciferase reporter assay was performed. Results showed that the activity of the firefly luciferase coding wild-type PPAR*α* 3′-UTR was significantly suppressed through cotransfection with miR-518d-5p mimics (Figure 2(b)), indicating that miR-518d can inhibit the expression of PPAR*α* via directly binding to the target sites in PPAR*α* 3′-UTR. To further explore the regulation mechanism of miR-518d on PPAR*α* and its downstream target genes, RT-PCR and Western blotting were performed. It was found that miR-518d downregulates the mRNA (Figure 2(c)) and protein (Figure 2(d)) expressions of PPAR*α*, CD36, ACO, and UCP2, suggesting the negative regulation of miR-518d on PPAR*α* and its downstream genes.

### 3.3. miR-518d Negatively Regulates PPAR*α*-Mediated Anti-Inflammatory Responses in HTR8/SVneo

**Figure 3:**
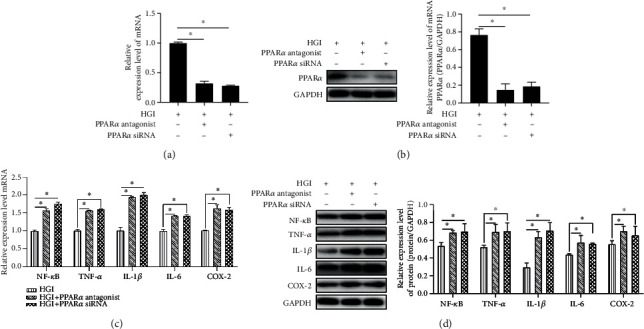
miR-518d negatively regulates PPAR*α*-mediated anti-inflammatory responses in HTR8/SVneo cells. Expression levels of PPAR*α* mRNA and protein were detected by (a) RT-PCR and (b) Western blotting. Expressions of mRNA and proteins of inflammatory factors were examined by (c) RT-PCT and (d) Western blotting, respectively. All data were represented as mean ± SD. ^∗^*p* < 0.05.

To further investigate the correlation between miR-518d and PPAR*α*, a PPAR*α*-specific antagonist and PPAR*α* siRNA interference were applied to the HGI group, respectively, resulting in the inhibition of PPAR*α* mRNA (Figure 3(a)) and protein (Figure 3(b)) expression levels. Contents of inflammatory factors were reduced with the inhibition of miR-518d. By blocking the PPAR*α* signalling pathway, we tested the expressions of genes (Figure 3(c)) and proteins (Figure 3(d)) of related inflammatory factors; results showed a significantly increasing trend after blocking the PPAR*α* pathway, suggesting that inhibiting miR-518d can activate the anti-inflammatory responses through the PPAR*α*-mediated pathway.

### 3.4. Regulatory Mechanisms of miR-518d through the PPAR*α*-Mediated NF-*κ*B Pathway in HTR8/SVneo

**Figure 4:**
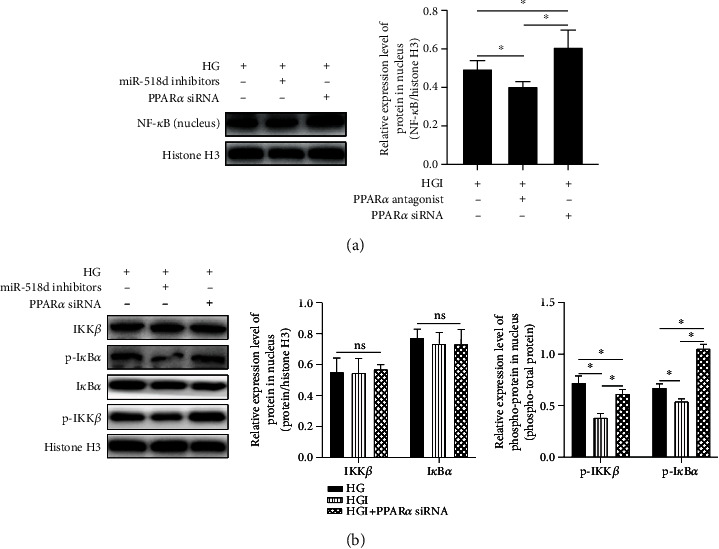
Regulatory mechanisms of miR-518d through the PPAR*α*-mediated NF-*κ*B pathway in HTR8/SVneo cells. (a) Expression levels of NF-*κ*B in the cell nucleus and (b) pathway-related proteins including the levels of phosphorylation detected by Western blotting. All data were represented as mean ± SD. ^∗^*p* < 0.05, ^*ns*^*p* > 0.05.

It was proven that miR-518d regulates the inflammatory responses by targeting PPAR*α* in the previous section. To further investigate whether miR-518d regulates the inflammatory responses through the NF-*κ*B signalling pathway by targeting PPAR*α*, PPAR*α* was knocked down in this study. The expression level of NF-*κ*B in the cell nucleus was detected using Western blotting; results showed that the level of NF-*κ*B in the nucleus was elevated at high glucose level, reduced with the application of miR-518d inhibitors, and was increased with the knockdown of PPAR*α* (Figure 4(a)). Further investigation on the expression levels of NF-*κ*B nuclear transport process-related proteins, IKK*β* and I*κ*B*α*, was carried out. It was shown that the high level of glucose can promote the phosphorylation of IKK*β* and I*κ*B*α*, while miR-518d inhibitors suppressed the phosphorylation. The pathway was activated with the induction of PPAR*α* knockdown (Figure 4(b)), suggesting that miR-518d promotes the NF-*κ*B nuclear transport process to regulate the inflammatory responses.

### 3.5. Regulation of miR-518d through the PPAR*α*-Mediated NF-*κ*B Pathway in GDM Mice

**Figure 5:**
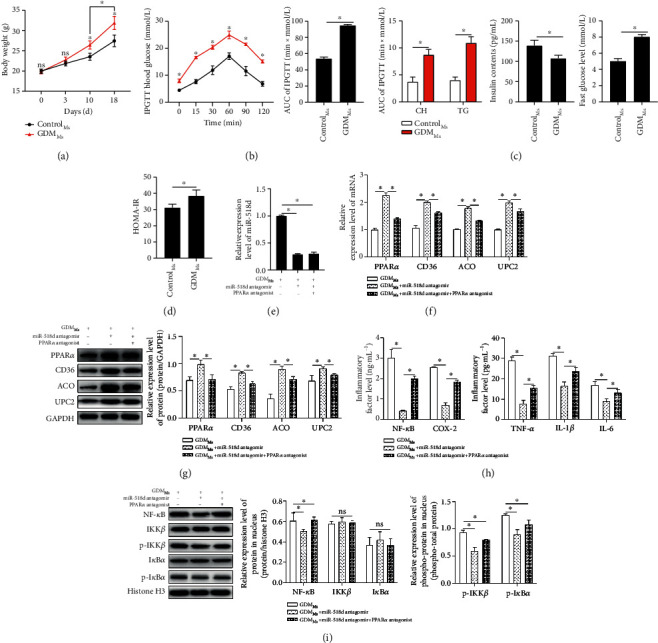
Regulation of miR-518d through the PPAR*α*-mediated NF-*κ*B pathway in GDM mice. (a) Body weights of mice in both Control_Ms_ and GDM_Ms_ groups recorded on the 0, 3, 10, and 18 days after pregnancy. (b) IPGTT on the 18^th^ day of pregnancy after 16-hour fasting. (c) Pathological indicator (CH, TG, FBG, and INS) tests on the 18^th^ day of pregnancy after 12-hour fasting. (d) HOMA-IR calculated based on the data obtained from pathological tests. (e) Expression of miR-518d after transfection. Expression levels of mRNA and proteins of PPAR*α* and its downstream genes detected by (f) RT-PCT and (g) Western blotting, respectively. (h) Contents of relative inflammatory factors (NF-*κ*B, TNF-*α*, IL-1*β*, IL-6, and COX-2) in peripheral plasma examined by ELISA. (i) Relative expressions in the nucleus of NF-*κ*B and pathway-related proteins detected by Western blotting. All data were represented as mean ± SD. ^∗^*p* < 0.05, ^*ns*^*p* > 0.05.

An *in vivo* study was performed to further validate the above findings with the establishment of GDM mouse models. The body weights of the GDM group were significantly higher than those with normal pregnancies on the 10^th^ and 18^th^ day (Figure 5(a)). In the intragroup comparison of the GDM mouse group, the average body weight on the 18^th^ day was remarkably higher than that on the 10^th^ day (Figure 5(a)). In the IPGTT, glucose levels achieved peak values at about 1 hour, and then, the levels went down in both groups of mice. However, results of IPGTT showed a higher peak value in the GDM group compared with the control group (Figure 5(b)). The increased levels of CH, TG, and GLU and the decreased level of INS were detected in mice with GDM (Figure 5(c)), leading to an elevation of HOMA-IR (Figure 5(d)), reporting a successful establishment of GDM mouse modelling. The miR-518d antagomir and the combined treatment of the antagomir and PPAR*α* inhibitors were then administered via tail veins in the GDM group, respectively. Expression of miR-518d was detected to validate the transfection efficiency (Figure 5(e)). RT-PCR and Western blotting were performed to detect the expression levels of PPAR*α* and its downstream genes. RT-PCR results of miR-518d and PPAR*α* expression levels are shown in Figure 5(f), and the relative expressions of PPAR*α*'s downstream proteins were examined by Western blotting (Figure 5(g)). These results agreed with that in the *in vitro* study previously and further proved that miR-518d negatively regulates PPAR*α* and its downstream genes. With the administration of the miR-518d antagomir, the inflammatory responses were notably withdrawn (Figure 5(h)). The nuclear transport process of NF-*κ*B was blocked by the miR-518d antagomir, followed by the weakened phosphorylation of NF-*κ*B pathway-related proteins, and the regulation of the miR-518d antagomir was reversely regulated by inhibiting PPAR*α* (Figure 5(i)). The above research further proved that miR-518d promotes the process of NF-*κ*B nuclear transport to regulate the inflammatory responses through targeting PPAR*α*.

## 4. Discussion

GDM is a disease that developed during pregnancy and leads to a high blood glucose level in the placenta [1]. Understanding of the related molecular mechanisms has great clinical significance on developing better therapeutic approaches for patients with GDM. It has been found that miRNAs are highly correlated with all aspects and stages involved in diabetes and its complications, including the clinicopathological process of GDM [5]. In this study, we cultured the human placenta trophoblast cell line in physiological and high glucose levels, respectively, transfected with miR-518d mimics and inhibitors, followed by the detections on the expression levels of PPAR*α*, its downstream genes, inflammatory factors, and NF-*κ*B pathway-related proteins to investigate the regulatory mechanisms of miR-518d on the development and progression of GDM through the PPAR*α*-mediated NF-*κ*B signalling pathway. We also successfully established mouse models with GDM to further prove the theoretical findings in cell culture.

Literatures have proved that relevant miRNAs play a vital role in the development and progression of GDM. We have detected that miR-518d is highly expressed in the placenta tissues of patients with GDM in contrast with those with normal pregnancies, along with the relatively high inflammatory responses observed in the placenta tissues and peripheral plasma when GDM occurs. It has been suggested that the inflammation responses in pregnancies are highly correlated with the occurrence of GDM, speculating that miR-518d may be involved in the relative regulations according to the diagnostic observations. Hence, the *in vitro* cell culture was carried out to validate the relationship between miR-518d and the expression levels of inflammatory factors. Transfecting with miR-518d inhibitors significantly reduced the expression levels of TNF-*α*, IL-1*β*, IL-6, and COX-2 in HTR8/SVneo, and the overexpression of miR-518d could promote the relative expressions, demonstrating that miR-518d is associated with the inflammatory responses.

PPAR*α* was validated as the target gene of miR-518d by performing the dual-luciferase reporter assay. The mRNA and protein expression levels of PPAR*α* and its downstream genes were decreased when the contents of miR-518d were elevated, suggesting that PPAR*α* is negatively regulated by miR-518d, and the development of GDM might be correlated with the obstruction of the PPAR*α* signalling pathway. As a primary nuclear transcription factor protein, NF-*κ*B was reported to be involved in the cell inflammation reactions [14]. Accumulated evidence strongly suggested that the ligand of PPAR*α* could negatively regulate the NF-*κ*B pathway, triggering the anti-inflammatory response. When inflammation occurs, the extracellular inflammatory signalling factors bind to receptors on the cell membrane and initiate a series of intracellular NF-*κ*B pathway downstream reactions. The receptor protein activates IKK*β* after being stimulated. The activated IKK*β* can phosphorylate the serine at the regulatory site of the I*κ*B*α* subunit of the NF-*κ*B-I*κ*B complex in the cell; hence, the I*κ*B subunit is modified by ubiquitination and then degraded by proteases to release the NF-*κ*B dimer leading to the anti-inflammatory reactions [15]. Therefore, after the administration of miR-518d inhibitors and PPAR*α* siRNA interference, the nuclear transport process of NF-*κ*B was blocked, along with the reductions of phosphorylated proteins. In summary, the inflammatory responses can be relieved through repressing the NF-*κ*B activity, indicating that the possibilities of relieving GDM can be achieved via suppressing the regulatory mechanisms of miR-518d, leading to an enhanced level of PPAR*α* and a negligible activity of NF-*κ*B.

In this study, we also carried out the *in vivo* experiments on mice to further support the conclusions obtained in the previous *in vitro* experiments. GDM mouse models were established successfully as described previously in GDM Mouse Model Establishment. We detected the relevant diagnostic indicators (body weights, IPGTT, CH, TG, GLU, and INS), followed by the evaluation of HOMA-IR. It is suggested that NF-*κ*B can promote the development of GDM through interacting with TNF-*α*, leading to an increased insulin resistance in GDM. It is reported that the expression levels of GLUT1 and GLUT4 could be repressed with the high content of miR-518d in the placenta tissues in mice with GDM, indicating that the glucose transport and insulin sensitivity might be downregulated with miR-518d leading to the glucose level imbalance and GDM occurrence [16]. The miR-518d antagomir and its combined treatment with GW6471 were separately injected into GDM mice via tail veins. It was found that miR-518d negatively regulates the expressions of PPAR*α* and its downstream proteins, promotes the inflammatory responses, and stimulates the intracellular NF-*κ*B signalling pathway leading to the development and progression of GDM. In summary, we have proven that the occurrence of GDM is regulated by the content level of miR-518d, and inhibiting its expression can relieve the inflammatory responses in placenta tissues which has a great clinical significance on diagnosing and treating GDM to rescue the pregnant women with GDM and prevent the offspring from having diabetes in their later life.

## Data Availability

The datasets used and/or analyzed during the current study are available from the corresponding author on reasonable request.
